# COMIT: identification of noncoding motifs under selection in coding sequences

**DOI:** 10.1186/gb-2009-10-11-r133

**Published:** 2009-11-20

**Authors:** Deniz Kural, Yang Ding, Jiantao Wu, Alicia M Korpi, Jeffrey H Chuang

**Affiliations:** 1 Department of Biology, Boston College, 140 Commonwealth Avenue, Chestnut Hill, MA 02467, USA

## Abstract

Coding nucleotide sequences contain myriad functions independent of their encoded protein sequences. We present the COMIT algorithm to detect functional noncoding motifs in coding regions using sequence conservation, explicitly separating nucleotide from amino acid effects. COMIT concurs with diverse experimental datasets, including splicing enhancers, silencers, replication motifs, and microRNA targets, and predicts many novel functional motifs. Intriguingly, COMIT scores are well-correlated to scores uncalibrated for amino acids, suggesting that nucleotide motifs often override peptide-level constraints.

## Abstract 2

COMIT is presented; an algorithm for detecting functional non-coding motifs in  coding regions, separating nucleotide and amino acid effects.

## Background

Over the past few years, coding nucleotide sequences have been shown to contain a myriad of functions independent of their encoded protein sequences [1]. Synonymous sites (sites in coding sequence that can be changed without altering the encoded amino acid sequence) that influence RNA localization [2], translation efficacy [3], mRNA splicing [4], mRNA stability [5], accessibility to the translation machinery [6], or even the structure of the folded protein [7] have been found. Meanwhile, theoretical studies have shown that the genetic code is optimal for the inclusion of noncoding functional signals within genes [8]. Such findings suggest that a tremendous amount of noncoding functional information may be contained in coding sequences. Sequences functional at the nucleotide level could be of critical importance for post-transcriptional regulation, which remains poorly understood [9-11]. However, computational methods, and in particular motif-detection methods, to identify such functions are lacking. In this work we present a novel approach to detect functional motifs in coding sequences using sequence conservation, solving the problem of how to separate noncoding from protein-coding effects, and we investigate the implications for eukaryotic gene regulation.

To detect noncoding functional signals, the associated conservation signatures must be distinguished from those engendered by the amino acid sequence. A classic method has been to separate cross-species substitution rates into the synonymous substitution rate *K*_*s *_[1,12] and the nonsynonymous substitution rate *K*_*A*_, with low *K*_*s *_values indicating the presence of noncoding selection [13]. However, *K*_*s *_measurements have typically been evaluated on complete genes, an approach that does not provide information about recurrent sequence motifs. Application of *K*_*s *_methods to motifs is hampered by the variable codon frame problem - namely, that instances of a sequence motif occur in varying codon frames in codons for a variety of amino acids. Also, different motifs will in general have different abundances. Because of this, for a fixed *P*-value each motif will have a different threshold deviation from the genome-average *K*_*s*_. This prevents one from effectively evaluating a motif based solely on its *K*_*s*_.

Some methods to detect unusually conserved motifs in intergenic sequence exist (for example, [14-16]), but because they do not account for the amino acid sequence they are fundamentally inappropriate for coding regions. A few studies of motif conservation have attempted to correct for the amino acid sequence [17,18], but these have been limited in scope. For example, Goren *et al*. [18] reported a method of identifying conserved dicodons, a special case that ignores the vast majority of motifs subject to the variable codon frame problem. Forman *et al*. [17] devised a detection algorithm that does not penalize nonconserved copies of a motif, hindering its applicability for motifs with large numbers of both conserved and nonconserved instances. The algorithm also requires conservation across 17 species, making it unsuitable for lineage-specific analysis, despite evidence that much gene regulation is likely to be lineage-specific [19,20].

In this work, we present a rigorous, novel computational algorithm, COMIT (for Coding Motif Identification Tool), to identify noncoding motifs in coding sequences using sequence conservation that overcomes the limitations of previous approaches. COMIT calculates a z-score of sequence conservation for each motif, corrects for the amino acid sequence in each species, and solves the variable codon frame problem. The z-score takes into account both conserved and non-conserved instances, allowing one to distinguish unusual motifs from as few as two species. To illustrate the power of the approach, we compare COMIT motif scores to maximum likelihood *K*_*s *_values, which we calculate for each motif based on the classic Li method originally designed for genes [21].

Application of COMIT reveals large numbers of noncoding motifs under natural selection in mammalian coding sequences. These results are robust - motifs with strong COMIT conservation scores also show strong sequence conservation via *K*_*s*_. In addition, each motif's conservation in one mammalian lineage strongly correlates with its conservation in others, which we demonstrate among the mouse-rat, human-dog, and elephant-tenrec lineages. Intriguingly, comparison of COMIT scores to scores calculated without calibrating for amino acids suggests that noncoding motifs can often overrule peptide-level constraints.

COMIT conservation scores have strong quantitative agreement with diverse experimental assays. For experimentally tested exonic splicing enhancer (ESE) motifs, we observe a clear correlation (Spearman ρ = 0.422) between COMIT score and splicing enhancer activity, and this is far superior to the correlation found when using *K*_*s *_(ρ = -0.0725). This ability to detect splicing motifs is remarkable, given that COMIT uses no information about splice boundaries. Exonic splicing silencers show intermediate negative conservation, consistent with natural selection acting to remove such sequences from coding regions. In addition, 21 of 24 hexamer submotifs of the ACS DNA replication origin motif in yeast have a positive COMIT score. Finally, microRNA binding motifs in both plants and animals exhibit higher COMIT scores, and some of the n-mers with the strongest overall conservation correspond to known microRNA binding motifs.

COMIT provides a practical, effective means to detect noncoding motifs in coding regions based on sequence conservation. Much remains to be discovered about splicing, RNA-protein binding, microRNA binding, and the diverse other possible noncoding functions in coding regions. Our studies with COMIT indicate that motifs relevant to these functions are likely to be common in eukaryotic coding sequences, and that many may be even more important than the amino acid sequences. We expect that COMIT will be a valuable tool for investigating such motifs in future studies.

## Results

### COMIT identifies an excess of highly conserved noncoding motifs in coding regions

**Figure 1:**
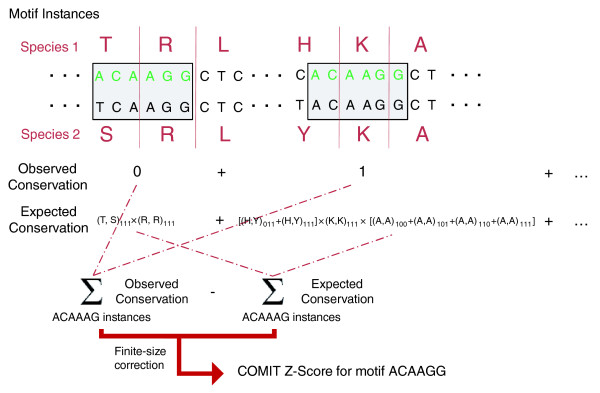
Schematic of the COMIT algorithm for identifying unusually conserved motifs in coding regions. The example illustrates how the score would be calculated for the motif ACAAAG, using genome-wide coding sequence alignments for two species. Each instance of the motif is identified in species 1, and the observed conservation - that is, whether all bases are identical among the two species - is calculated. The expected conservation at each instance is modeled from genome-wide frequencies of nucleotide-level conservation patterns conditional on the aligned amino acids. For each instance, the expected conservation is calculated from all possible ways in which the motif could be conserved at that location given the amino acids in each species, using values from Table 1 (typically some of these quantities, such as (H, Y)_111_, will be zero). The observed and expected conservation levels are compared and normalized to yield a conservation z-score for each motif.

Using alignments of all mouse and human coding sequences, we calculated a COMIT z-score for the sequence conservation of all 4,096 6-mers. For each motif, we considered every instance in which it occurred in the coding regions of human, measured the number of conserved instances, and compared this to the number of conserved instances that would be expected given only the amino acid alignments. A schematic of this procedure is shown in Figure 1, with a full description provided in the Materials and methods. Out of these 4,096 motifs, we found 503 with a z-score > 15, suggesting that many motifs are subject to noncoding pressures. In contrast, one would expect < 10^-46 ^motifs to have z > 15 in a normal distribution. We performed a similar evaluation of motifs in the *Saccharomyces cerevisiae*- *Saccharomyces bayanus *comparison. For these yeasts we found 115 motifs with z > 10, compared to < 10^-19 ^expected, suggesting that yeast species contain many motifs under noncoding pressures in coding regions as well. Prokaryotes also exhibited an excess of motifs with strong conservation. When we applied COMIT to aligned *Escherichia coli *and *Yersinia pestis *coding regions, we found 17 hexamers with z > 20 and none with z <-10.

Z-scores were robust to the choice of species used to define motif instances. For example, the mouse-human results described above were based on instances matching the motif in the human lineage. We also measured z-scores using the motif instances in the mouse lineage and found the z-scores under these two definitions to be extremely similar (Spearman correlation ρ = 0.971, *P*-value < 0.00001).

**Figure 2:**
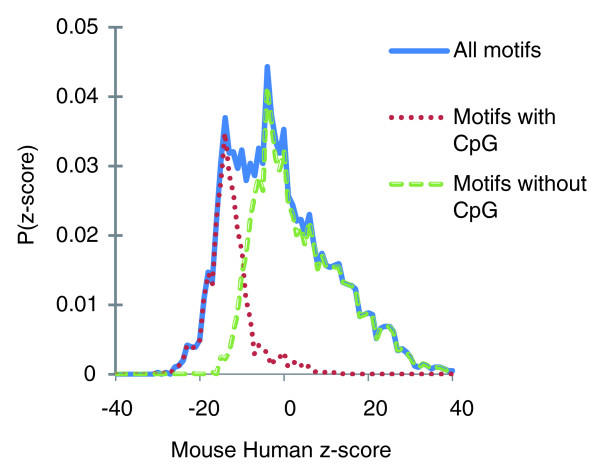
Distribution of mouse-human COMIT z-scores. Motifs containing CpGs have systematically lower conservation scores (dotted red curve, mean = -12.8), while the remaining motifs have higher conservation scores (dashed green curve, mean = 5.2), consistent with hypermutability of CpG dinucleotides in mammalian genomes. The non-CpG distribution has an excess of motifs with high z-scores, as can be seen from the long rightward tail. This suggests that selection has acted to maintain sequence conservation of these motifs across species.

The distribution of mammalian z-scores can be seen in Figure 2. The shape of the distribution is wider than that of a normal distribution, which likely reflects mutational influences such as regional substitution rates, regional composition preferences, and so on. A key predictive variable appears to be whether or not a motif contains a CpG. Motifs containing CpGs have systematically lower conservation scores (dotted red curve, mean = -12.8), while the remaining motifs have higher conservation scores (dashed green curve, mean = 5.2), and none of the motifs with z > 15 from the mouse-human comparison contain a CpG. This behavior is consistent with the known hypermutability of CpG dinucleotides in mammalian genomes.

The shape of the non-CpG motif distribution suggests that selection has increased the conservation of a number of motifs. The non-CpG distribution has an excess of motifs with high z-scores, as can be seen from the long rightward tail extending out to z ~ 40. In contrast, the distribution decays to zero on the left at z ~ -17. A simple explanation is that motifs with very large z-scores have been influenced by selection.

### COMIT motifs are robust with maximum likelihood K_s _measures

To verify the robustness of motifs predicted by the z-score method, we implemented two maximum likelihood methods for calculating the synonymous substitution rate *K*_*s *_from aligned codons, based on the classic Li algorithm for calculating *K*_*s *_for a gene (see Materials and methods). These methods give *K*_*s *_values for each motif, providing a comparison for the z-score results.

The first is a naïve codon completion method, in which we calculated *K*_*s *_values for each motif based on the full codons that overlap any instance of the motif. Although this method contains noise due to the naïve completion of codons, it has the advantage of being easily implemented using PAML [22]. The second is a nucleotide-by-nucleotide method that solves the noise issues. This algorithm was implemented independently of PAML. In comparing the *K*_*s *_and z-score results, we expected that motifs with strong conservation z-scores would have low *K*_*s *_values.

**Figure 3:**
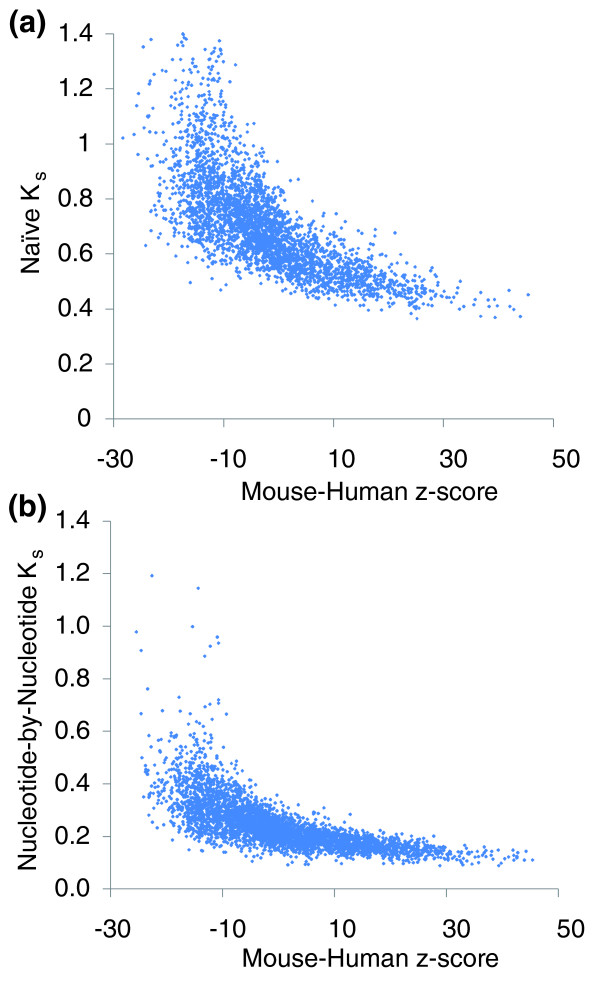
Comparison of COMIT z-scores to maximum likelihood *K*_*s *_scores. There is a clear correlation between mouse-human z-scores and mouse-human *K*_*s *_based on **(a) **naïve codon completion or **(b) **nucleotide-by-nucleotide *K*_*s*_. These correlations indicate that the qualitative conservation of many motifs is not method-dependent.

We first compared the motif z-scores to the *K*_*s *_values from the naïve codon completion method. Figure 3a shows the *K*_*s *_values for each motif calculated from human-mouse alignments compared to the z-score values for each motif for human-mouse. We observed a clear correlation between the z-scores and *K*_*s*_.

We next compared the mouse-human z-scores to the mouse-human nucleotide-by-nucleotide *K*_*s *_values (Figure 3b). Again, we observed a strong correlation between the motif z-scores and the *K*_*s *_values. This correlation is even sharper when the larger human-dog-rat-mouse phylogeny is analyzed (Figure S1 in Additional data file 1). Figure 3 also illustrates that the nucleotide-by-nucleotide *K*_*s *_is a better measure than the naïve codon completion method. At any given z, the distribution of *K*_*s *_values for the nucleotide-by-nucleotide method is narrower than that for the naïve codon completion, and consequently the correlation with z-scores is stronger (see also Figure S2 in Additional data file 1).

These comparisons indicate that the essential motif behaviors predicted by COMIT are not method-dependent. However, this does not mean the methods are interchangeable. COMIT has two important advantages over *K*_*s *_methods. One is that the z-scores compensate for copy-number stochasticity while the *K*_*s *_values do not. A second is that the z-scores have a much broader range of values than the *K*_*s *_scores, making the z-scores more informative for distinguishing unusual motifs even empirically.

### Motif conservation is robust across mammalian lineages

**Figure 4:**
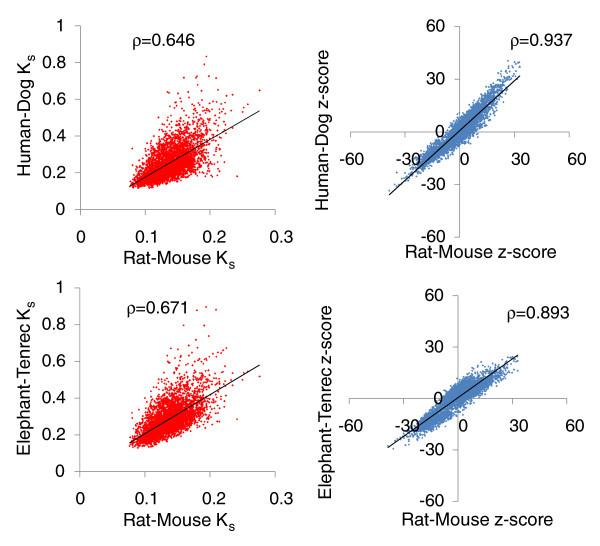
Motif conservation is robust across the rat-mouse, human-dog, and elephant-tenrec lineages. This is visible when using nucleotide-by-nucleotide *K*_*s *_values, or when using COMIT z-scores. Strong Spearman rank correlations (ρ) are observed in comparisons of both (human-dog)-(rat-mouse) and (elephant-tenrec)-(rat-mouse). Spearman correlations are considerably stronger for the COMIT z-scores, indicating the superiority of the method to *K*_*s*_. For each of these comparisons the Spearman correlation is highly significant, with permutation test *P*-value < 0.00001.

We next compared the behavior of motifs in separate mammalian lineages. Figure 4 compares the nucleotide-by-nucleotide *K*_*s *_in pairs of independent mammalian lineages (rat-mouse, human-dog, elephant-tenrec), as well as the z-scores in these lineages.

Motifs behave very similarly in the rat-mouse, human-dog, and elephant-tenrec lineages. For example, the correlation in *K*_*s *_values between the rat-mouse and human-dog lineages is highly significant (Spearman ρ = 0.646, permutation test *P*-value < 0.00001), and the correlation between the rat-mouse and elephant-tenrec lineages is similar (ρ = 0.671, *P*-value < 0.00001). These correlations are even stronger for the z-scores: (rat-mouse versus human-dog ρ = 0.937, *P*-value < 0.00001) and (rat-mouse versus elephant-tenrec ρ = 0.893, *P*-value < 0.00001). This suggests that motifs are under similar pressures among different branches of the mammalian lineage.

There is especially strong agreement in the sets of motifs with high conservation scores, and which are hence likely to be under selection. The numbers of motifs with z > 15 in each of the three lineages are (rat-mouse, 306; dog-human, 363; elephant-tenrec, 98). If these sets were independent, one would expect 306 × 382 × 98/(4,096^2^) = 0.7 motifs to have z > 15 in all three lineages. However, there are actually 82 such motifs, and each of these has z > 15 in the mouse-human comparison as well. On average, each of these motifs has approximately 2,100 more conserved instances than would be expected by chance (each motif occurs on average 19,000 times). Such motifs are excellent candidates for having previously uncharacterized functions.

### COMIT explains the activity of diverse experimentally tested motifs

#### Exonic splicing enhancers

**Figure 5:**
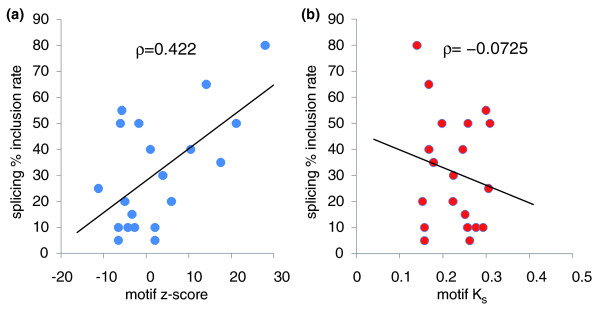
Experimentally verified ESEs are preferentially conserved by natural selection. **(a) **Motif z-scores (greater z indicates greater conservation), based on mouse-human comparisons, show a strong quantitative correlation (Spearman ρ = 0.422, permutation test *P*-value = 0.045) with splicing inclusion rates as measured experimentally in [4]. **(b) ***K*_*s *_values, also based on mouse-human comparisons, show a far weaker correlation (ρ = -0.0725, *P*-value = 0.606). Black lines indicate regression fits. While the two motifs with the highest splicing inclusion rates do exhibit below-average *K*_*s *_values, this is the only apparent effect, indicating that COMIT scores are better for assessing functional motifs.

To verify the efficacy of our algorithms, we examined the sequence conservation of 20 hexamer coding motifs whose ESE activity has been measured experimentally [4]. We observed a clear correlation (ρ = 0.422, *P*-value = 0.045) between human-mouse z-scores and the quantitative ESE activities, as measured by the splicing inclusion rates engendered by the motifs (Figure 5a). This correlation shows that COMIT z-scores can not only identify functional motifs but also predict their activity level. In contrast, K_s _values are much less predictive of splicing inclusion rates. Figure 5b shows the splicing inclusion rates for these motifs as a function of their K_s _in the mouse-human phylogeny. The correlation is far weaker (ρ = -0.0725, *P*-value = 0.606). In fact, even when the phylogeny is extended to the mouse-rat-human-dog phylogeny, the correlation of K_s _(as measured by the total branch length in the phylogeny) to splicing inclusion rates is only ρ = -0.246 (*P*-value = 0.867). This is less informative than the z-scores from just the mouse-human comparison.

This agreement with the splice enhancer experiments was an unexpectedly strong result, given that our conservation z-score used no experimental information other than coding DNA alignments. In contrast, Fairbrother *et al*. [4] chose these motifs for testing based on more detailed criteria, involving motif frequency comparisons in exons, introns, exons with clear terminal splice signals, and exons without clear terminal splice signals. Nevertheless, our z-score method rated the 20 motifs similarly as the Fairbrother *et al*. method. Of the 20 tested motifs, Fairbrother *et al*. had predicted that ten would have splice enhancer activity, and we found that eight of ten of these had positive COMIT scores. They had predicted that the remaining ten would not have enhancer activity, and of these only one had a positive COMIT score.

To further validate our predictions, we compared the mouse-human conservation z-scores to a set of experimental splicing inclusion rates associated with 16 octamer motifs, as measured by [23]. We observed good agreement with experimental splicing inclusion rates, though it was necessary to consider CpG-containing motifs separately. We initially measured the correlation between z-score and splicing inclusion rate for these 16 motifs, finding a small correlation (ρ = 0.0854, *P*-value = 0.363). However, seven of the motifs contain CpG dinucleotides. These CpG-containing motifs exhibit systematically lower conservation rates, with all seven having z-scores below zero. CpG effects were not an issue for the Fairbrother *et al*. [4] set because none of those hexamers contain CpG dinucleotides. For the Zhang and Chasin dataset [23], when we ignored the CpG-contaning motifs we recapitulated a strong correlation between splicing inclusion rate and z-score (ρ = 0.753, *P*-value = 0.013; see Discussion for a more detailed consideration of CpG effects).

#### Exonic splicing silencers

**Figure 6:**
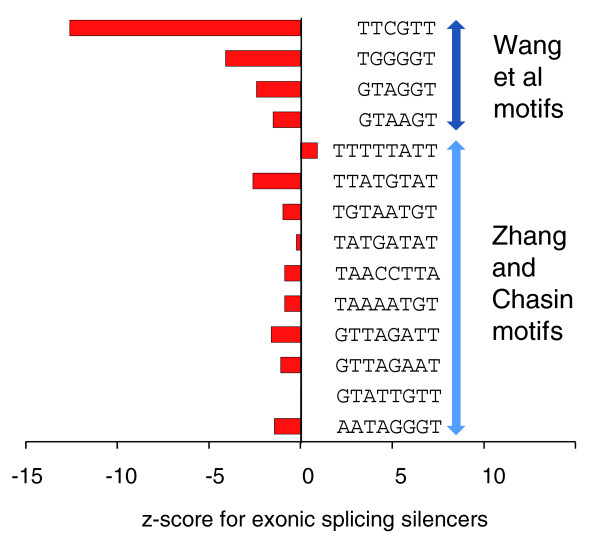
Mouse-human z-scores of motifs with experimentally verified ESS activity, which is deleterious for exons. Four of four of the Wang *et al*. [24] experimentally verified ESSs have negative z-scores, consistent with negative selection acting to remove them from coding regions. For the Zhang and Chasin [23] experimentally verified ESSs, nine of ten have negative z-scores (GTATTGTT, z = -0.006).

We next analyzed experimental data on exonic splicing silencers (ESS) from Wang *et al*. [24]. ESSs are deleterious for genes and are subject to negative selection. Using a green fluorescent protein reporter assay, they identified four hexamers with ESS activity clearly greater than that of control hexamers (Figure 4a of [24]). We found that all four of these had negative mouse-human z-scores (Figure 6: TTCGTT, -12.6; GTAAGT, -1.5; TGGGGT, -4.1; GTAGGT, -2.4). Thus, the z-score method is also capable of detecting motifs under negative selection. One of these motifs, TTCGTT, has a CpG, which explains its very low z-score, though the CpG effect is probably not sufficient to explain such a low value (see Discussion). For the non-CpG-containing ESS motifs, the magnitudes of the z-scores are not as large as those of the ESEs (compare to Figure 5a). This is reasonable, since extreme negative selection would tend to remove copies of the motif from each genome, rendering the motif invisible to a sequence conservation algorithm. For this reason, one would expect motifs under negative selection to have moderate, rather than extreme, negative z-scores - which is what is observed. We observed similar behavior for motifs tested in separate splicing silencer experiments by Zhang and Chasin [23]. For octamer motifs with clear splicing silencer activity (> 50%), we observed that nine out of ten had negative z-scores. CpG dinucleotides are not responsible for these low z-scores, as none of the octamer motifs contain a CpG.

#### DNA replication origins

We next examined the conservation of a DNA-level motif involved in yeast DNA replication known as the ACS motif. Nieduszynski *et al*. [25] identified this motif based on phylogenetic conservation and experimentally verified it at 228 *S. cerevisiae *intergenic replication origins. Nieduszynski *et al*. reported being unable to phylogenetically evaluate ACS motifs in coding regions due to interference from the amino acid signal. Because of this it has been uncertain whether instances of the ACS motif in coding regions are active, though it is worth noting that protein-coding regions make up approximately 70% of the *S. cerevisiae *genome [26].

**Figure 7:**
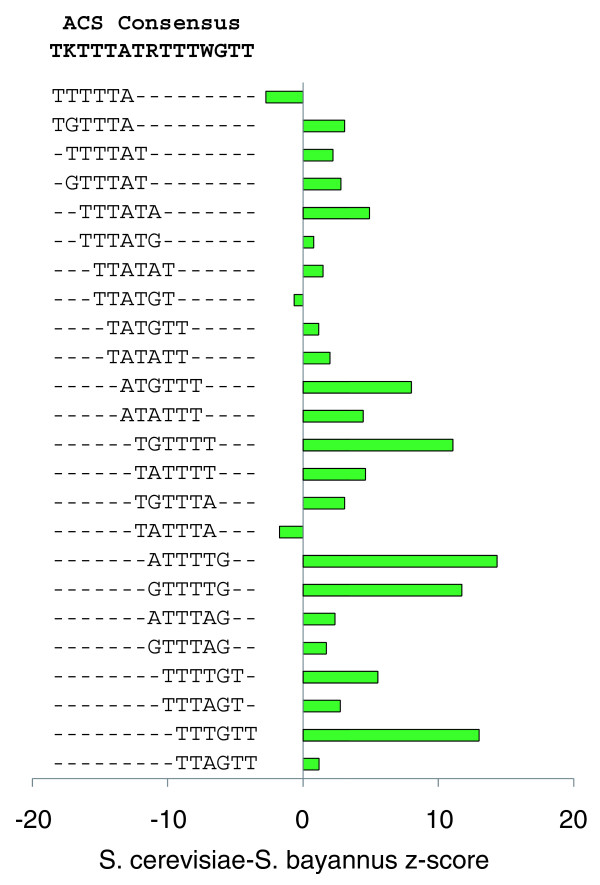
Conservation of hexamer submotifs of the yeast ACS DNA replication origin motif. Of the 24 hexamers, 21 are consistent with the ACS consensus TKTTTATRTTTWGTT and have positive z-scores in comparisons of *S. cerevisiae *and *S. bayanus *coding sequences. These results support the hypothesis that ACS motifs in coding regions are functionally active, and also indicate that COMIT is capable of detecting coding motifs functional at the DNA level.

COMIT gives consistently positive scores for the ACS motif in coding regions. We tested the z-scores of all 6-mers that coincide with this motif, given the degenerate consensus TKTTTATRTTTWGT. We found that 21 of 24 6-mers have positive z-scores based on coding sequence alignments of *S. cerevisiae *and *S. bayanus *(Figure 7). These results support the hypothesis that ACS motifs in coding regions are functionally active and suggest that COMIT is capable of detecting coding motifs functional at the DNA level.

#### MicroRNA binding motifs

Finally, we considered whether COMIT was able to detect microRNA binding sites in coding regions. We first examined the *Oryza sativa *(rice)-*Arabidopsis thaliana *COMIT scores of motifs that would complement 8-mer tilings of known microRNAs from these species. Eight-mers complementary to plant microRNAs have higher average COMIT scores than the overall set of 8-mers (μ_1 _= 0.90, μ_2 _= 0.096; μ_1 _= μ_2_, *P *= 3.6e-09 Welch's *t*-test), consistent with microRNA binding in plant coding regions. We next examined microRNA binding in animal coding regions. While animal untranslated regions have been studied extensively for microRNA binding, animal coding regions have only recently been recognized as potentially important for microRNA targeting [27]. We found that sites complementary to microRNA 7-mer seed sequences [28] have significantly higher mouse-human COMIT scores than the overall set of 7-mers (μ_1 _= 4.18, μ_2 _= -0.31; μ_1 _= μ_2_, *P *= 1.2e-12). Of the 156 curated animal 7-mers, 107 have z > 0, and 12 of 156 have z > 15. These results suggest that many mammalian microRNAs bind in coding regions.

## Discussion

In this work we present COMIT, a novel algorithm to detect motifs with noncoding functions in coding regions. The COMIT z-scores provide a practical statistic for identifying unusually conserved motifs, with the scores corrected for copy number stochasticity and exhibiting a broad range of values. Although *K*_*s*_-based analyses have been useful for studies of the behavior of large groups of motifs [29,30], *K*_*s *_is not precise enough to analyze individual motifs. This is clear from the much weaker correlations of *K*_*s *_versus splicing enhancer activity when compared to COMIT scores versus splicing enhancer activity. Meanwhile, the strongly conserved motifs identified by COMIT are robust in different mammalian lineages. Such motifs, for example, the 82 with z-score > 15 in mouse-rat, human-dog, and elephant-tenrec, constitute some of the most promising candidates for novel functions in mammalian coding regions. While we have focused primarily on mammals, COMIT is applicable to arbitrary pairs of species. COMIT scores for all hexamers in each of the mammalian, yeast, and prokaryote comparisons described in the manuscript are provided in Table S1 in Additional data file 2. *K*_*s *_values for motifs (Table S2 in Additional data file 2) and a list of the 82 highly conserved motifs described above (Table S3 in Additional data file 2) are also provided.

Our approach to detecting motifs used no information other than aligned coding sequences - making it remarkable that our predictions agree so well with the broad range of experimental data. One might speculate that this is because splicing and DNA replication are among the most important functions in the genome. However, there are many motifs with even larger z-scores, suggesting that COMIT can detect diverse types of biological functions. For example, the 82 hexamers with z > 15 in mouse-rat, human-dog, and elephant-tenrec are disjoint from the hexamers in the ESE set, and these 82 hexamers have, on average, 2,100 more conserved instances than would be expected based on the amino acid sequences alone. Although it is not obvious what threshold z-score should be used to classify a motif as functional (since the observed z-score distribution of Figure 2 deviates from a normal distribution and the null model is a simplification of neutral evolution), the fact that these 82 hexamers have stronger conservation than experimentally verified ESEs provides good evidence that they are under purifying selection.

Some of the highly conserved motifs correspond to known microRNA binding motifs, and the extreme conservation of known microRNA motifs suggests that other extremely conserved motifs may have previously unknown microRNA-binding function as well. For instance, in the mouse-human 8-mer data, the motif with the largest z-score is CTACCTCA (z = 23.5, 553 conserved instances, 241.4 expected by chance), which matches the *let-7 *microRNA binding site. Interestingly, Forman *et al*. [17] also detected this motif in their 17-way species comparison, but COMIT was able to find it using only two species.

COMIT's central concept is its isolation of nucleotide-level effects by conditioning on the amino acid sequences in each species, an approach different from previous ESE-detection approaches [4,23,31-34]. While not all nucleotide-level selection may be detected by COMIT, this null model is explicitly designed so that the COMIT scores are free of influence from amino acid effects. In contrast, Fairbrother *et al*. [4] identified unusual motifs by comparing motif frequencies in exons either with or without clear terminal splice signals. That approach gives a more ambiguous calibration for amino acid effects, as it depends on the assumption that the two exon groups have similar amino acid-level selection pressures. COMIT, on the other hand, directly calibrates for the amino acids in each species at every motif instance. This entails a different type of assumption, which is that COMIT's underlying null model is homogeneously applicable (this is an assumption about neutral synonymous codon usage along the genome, as opposed to an assumption about amino acid selection). Another contrast is provided by the algorithm of Forman *et al*. [17], which uses a null model that is conditioned on the codons overlapping a motif. Conditioning on codons leads to difficulties in the interpretation of scores, since the specification of a codon contains information about both the amino acid sequence and the nucleotide sequence. Under a codon-based null, a motif's score will be influenced by both amino acid pressures and nucleotide pressures, the balance of which is not *a priori *known.

The closest existing algorithm to COMIT is that of Goren *et al*. [18], which can be thought of as a special case of COMIT for motif instances overlapping exactly two full codons and with a null model conditioned on codons. Goren *et al*. reported 285 unusual motifs, and as expected these generally have high COMIT scores (average z = 11.2). However, there are some notable differences: 45 of the Goren *et al*. motifs have COMIT scores < 0, suggesting that codon frame may be important to some motifs. Also, the Goren *et al*. method cannot evaluate motifs containing stop codons in the canonical frame, such as TGATGA, because of that method's restriction to dicodons. Interestingly, COMIT suggests that TGATGA may be under selection when it occurs in other frames, as TGATGA has z > 7.9 in all mammalian comparisons, including z = 17.6 for mouse-human.

Combining COMIT with other analytical approaches should lead to more comprehensive understanding of the functions in coding sequences. Some motifs may be restricted to only certain loci, and detection of these would be aided by methods that consider motif overrepresentation. A few overrepresentation approaches have been applied to coding regions [2,4,35-39], though their agreement with experiment has been mixed. Locus-based approaches [40-42] also complement COMIT, although resolving individual motif instances with such approaches is still challenging.

### Dinucleotide considerations

COMIT's null model is conditioned only on the amino acid sequences, and other sequence influences such as amino acid-changing dinucleotide biases (dinucleotide biases that maintain an amino acid are accounted for in our null model) could be incorporated in a more sophisticated null model. Unfortunately, because dinucleotide biases are not independent of the amino acid sequences, it is difficult to include them without recoupling coding and noncoding behaviors. Other, probably less important, effects that we have not treated in the model include mutational heterogeneity along the genome [43] and location-specific codon usage bias [44].

We did test a simple model taking into account the best-known dinucleotide effect in mammals, CpG hypermutability. We recalculated z-scores for each motif, assuming that the CpG effect was so strong that the expected frame-specific conservation rate at a CpG site would be independent of the amino acid sequence (see Materials and methods). Under this model, one CpG-containing silencer motif was affected [24]: TTCGTT had a z-score change from -12.7 to -4.2, maintaining the expected negative selection. Seven CpG-containing splice enhancer motifs from the Zhang and Chasin data [23] showed altered (higher) z-scores. However, correcting for the CpG effect did not lead to a strong correlation of z and enhancer activity in the full Zhang and Chasin set (ρ = 0.181, *P*-value = 0.251). This indicates that CpG effects are subtler than this simple model. This is a notable limitation of COMIT, as 1,185 out of the 4,096 possible hexamers contain a CpG.

Incorporation of better parameterized (presumably neutral) dinucleotide effects [45,46] would be a valuable future goal. This is challenging because the strength of neutral dinucleotide biases has not been precisely quantified [47], and the development of methods to accurately account for dinucleotide biases is an active problem, even for motifs in noncoding sequences [48]. For these reasons, we have left dinucleotide biases out of the COMIT null model, and instead dealt with them at the stage of interpretation of scores. Nevertheless, the empirical agreement of COMIT scores with the multiple types of experimental data, especially when CpG-containing motifs are considered separately, demonstrates that the current implementation of COMIT is already useful for real functional motifs.

### Are noncoding pressures common in coding sequence?

The large number of motifs with strong conservation suggests that coding sequences could contain a considerable amount of sequence functional for noncoding reasons. Previous studies have shown that proteins can tolerate significant amino acid changes without inactivating the protein [49], supporting such a view. To further investigate this, we compared our motif scores to scores calculated without correcting for the amino acid sequence. We found these calibrated and uncalibrated scores to be highly correlated (ρ = 0.885, *P*-value < 0.00001, mouse-human comparisons for all hexamers). A plot of these scores versus one another is given in Figure S3 in Additional data file 1.

This strong correlation is consistent with the idea that a non-trivial fraction of the conservation in coding sequences is due to noncoding pressures, rather than amino acid pressures. Although some of this may be due to neutral dinucleotide biases not contained in our model, the high copy numbers of motifs with strong conservation scores across multiple mammalian species, together with the experimental validations, suggest that selection plays an important role. This supports more specific findings that nucleotide-level selection for splicing enhancer elements [50,51] and nucleosome positioning signals [52,53] are strong enough to influence protein sequences. These results indicate that the balance of pressures in coding sequence is more heavily tilted toward the nucleotide end than has been previously assumed.

## Conclusions

We have developed COMIT, a computational algorithm that effectively detects functional noncoding motifs in coding regions using sequence conservation. Our studies indicate that such motifs, which play key roles in post-transcriptional regulation or DNA-level functions, are common in mammalian genomes, and may often be more important than the amino acids with which they coincide. COMIT provides a valuable tool for identifying and comparing the functions in coding regions for arbitrary phylogenies.

## Materials and methods

### Datasets

Coding sequence alignments were obtained by identifying mutual-best-hit protein orthologs, CLUSTALW aligning the protein orthologs, and back-translating to the DNA level. Full details of the procedures for pairwise mammalian alignments are given in [54]. The four-species alignments of human, mouse, rat, and dog were obtained as described in [13]. Yeast alignments were obtained as described in [55]. Rice and *Arabidopsis *sequences were obtained from the The Institute for Genomic Research (TIGR) ftp site [56]. *E. coli *and *Y. pestis *data were obtained from the University of Wisconsin ASAP database.

### COMIT z-score for motif conservation

The COMIT z-score method detects unusually conserved motifs of arbitrary length and codon frame, properly correcting for the amino acid sequence in each species. To calibrate for the amino acids, we first tabulate the statistics of DNA conservation for all pairs of aligned amino acids, using all coding sequence alignment data between the two genomes. In particular, we use the aligned amino acid statistics to calculate the frequency of each of the 2^3 ^= 8 conservation patterns (000, 001, 010, 011, 100, 101, 110, 111, where 1 means a conserved base and 0 means a non-conserved base) for the three nucleotides underlying the aligned amino acids. This defines eight functions *f*_000_(*α*, *β*), *f*_001_(*α*, *β*), ..., *f*_111_(*α*, *β*) for the aligned amino acids *α*, *β*. These functions give the calibrated background probabilities of any bases in a codon being conserved, given the amino acids in each species (Table 1).

**Table 1:** Abridged table of mouse-human genome-wide codon conservation frequencies, as a function of each of the 20 × 20 pairs of aligned amino acids

AA1	AA2	#	000	001	010	011	100	101	110	111
F	F	308,260	0.000	0.000	0.000	0.000	0.000	0.000	0.202	0.798
F	S	3,951	0.028	0.042	0.000	0.000	0.337	0.593	0.000	0.000
F	T	716	0.457	0.543	0.000	0.000	0.000	0.000	0.000	0.000
F	N	220	0.377	0.623	0.000	0.000	0.000	0.000	0.000	0.000
F	K	160	1.000	0.000	0.000	0.000	0.000	0.000	0.000	0.000
.		.			.			.		.
.		.			.			.		.
S	F	3,660	0.022	0.050	0.000	0.000	0.322	0.607	0.000	0.000
S	S	616,045	0.004	0.003	0.000	0.000	0.000	0.000	0.302	0.691
.		.			.			.		.
.		.			.			.		.
.		.			.			.		.
G	R	7,924	0.000	0.000	0.393	0.607	0.000	0.000	0.000	0.000
G	G	521,714	0.000	0.000	0.000	0.000	0.000	0.000	0.327	0.673

For a given pair of amino acids, there are eight possible conservation patterns for the underlying nucleotides (000, 001, 010, 011, 100, 101, 110, 111), where 1 means a conserved base and 0 means a non-conserved base. These frequencies provide a null model for the expected conservation patterns at the nucleotide level, given the amino acid sequence. Here '#' indicates the number of instances in which amino acid 1 (AA1) is aligned to AA2 in the complete set of coding alignments between mouse and human.

To determine whether a motif is unusually conserved, we compare the actual number of conserved instances of the motif to the number expected based on the *f *functions. The full procedure is summarized in Figure 1. The expected number can be calculated by considering the *f *function values in the set of instances where the motif occurs. For example, suppose we are interested in a 6-bp motif in which one of its instances begins at the second position of a codon (right instance in Figure 1), overlapping amino acids *α*_1 _*α*_2_*α*_3 _in the first species and *β*_1_*β*_2 _*β*_3 _in the second species. Then the probability that this motif would be conserved by chance in this instance would be [*f*_011_(*α*_1_, *β*_1_) + *f*_111_(*α*_1_, *β*_1_)] × *f*_111_(*α*_2_, *β*_2_) × [*f*_100_(*α*_3_, *β*_3_) + *f*_101_(*α*_3_, *β*_3_) + *f*_110_(*α *_3_, *β *_3_) + *f*_111_(*α *_3_, *β *_3_) ]. The calculated quantity covers all possible ways in which the motif could be conserved at that location given the amino acids in each species. In Figure 1 we have used a shorthand notation. So, for example, (H, Y)_011 _in Figure 1 is equivalent to *f*_011_(*H*, *Y*) in the notation here.

These background conservation probabilities at each motif instance can be summed to give the total expected number of conserved instances for the motif. By comparing this sum to the observed number of conserved instances, we can identify motifs that have unusually high levels of conservation. An important property of the method is that it handles motifs occurring in any translation frame, unlike specialized methods that require motifs to exactly cover complete codons [18]. One notable benefit of this is that it allows one to evaluate motifs that are rare in one translation frame but not in others, by aggregating data from all translation frames together. For example, the motif TGACGA cannot occur in the first translation frame because TGA encodes a 'stop', but the motif occurs abundantly in the second and third translation frames.

To determine the COMIT score for a given motif, we use z-score statistics, which we and other groups have previously used to identify unusually conserved motifs in intergenic regions [15,26,57]. If *N *is the total number of instances of a motif, *N*_*c *_is the number of conserved instances, and *N*_*c*_(*exp*) is the number of expected conserved instances, then the z-score for a motif is given by:

The use of z-score statistics makes the method more sensitive as *N *increases, consistent with the idea that functionally irrelevant stochastic effects will more easily distort the conservation rates of low copy number motifs. The *P*-value of a positive z-score can be calculated from the expected normal distribution by integrating the area under a Gaussian from *z *to 8. This 'area-under-the-curve' approach is usual statistical practice, as compared to the 'height-of-the-curve' approach in the Forman *et al*. [17] method.

A version of COMIT has been implemented in Python and is available upon request.

### Z-score motif conservation without correction for amino acid sequence

For the uncalibrated z-score algorithm, z is again calculated as:

However, here *N*_*c*_(*exp*) is based on the fraction of all 6-mers conserved in the coding alignments without regard to the underlying amino acid sequences.

### Maximum-likelihood K_s _methods for motifs

Our *K*_*s *_methods are modified versions of Li's method [21], which accounts for multiple substitutions at each site. These methods are similar to calculations in [29,30] to calculate *K*_*s *_in segments of DNA, though we have adapted the procedure to handle arbitrary motifs. Briefly, the Li method calculates the maximum-likelihood number of synonymous substitutions between two sequences, noting transitional and transversional differences separately. The method is based on the parameters: *L*_*i *_(*i *= 0, 2 and 4) - the numbers of synonymous sites with degeneracy 0, 2, and 4, respectively, in the two sequences being compared; *S*_*i *_- the numbers of synonymous transitional differences in the two sequences being compared; and *V*_*i *_- the numbers of synonymous transversional differences. For cases where the two codons differ from each other in multiple positions, substitution paths are unweighted [12]. The formula for *K*_*s *_is given by:

where *A*_*i *_= 1/2 *ln*(*a*_*i*_) *- *1/4 *ln*(*b*_*i*_) and *B*_*i *_= 1/2 *ln*(*b*_*i*_)*; a*_*i *_= 1/(1 - 2*P*_*i *_- *Q*_*i*_) and *b*_*i *_= 1/(1 - 2*Q*_*i*_)*; *and *P*_*i *_*= S*_*i*_/*L*_*i *_and *Q*_*i *_*= V*_*i*_/*L*_*i*_.

Existing software, such as PAML [22], can calculate the Li synonymous substitution rate from codon-by-codon alignments of two sequences. However, PAML is not suitable for the calculation of *K*_*s *_values for motifs because different instances of a motif occur in different codon frames and in different amino acids. We devised two approaches to calculate an analog of the Li *K*_*s *_rate for motifs.

#### Naïve codon completion

As a first approach to a motif *K*_*s *_value, we calculated the Li substitution rate based on the complete codons overlapping at least one base of any instance of the motif. For example, suppose we have two aligned sequences containing motif TACCTC, where sequence 1 is: aTA|CCT|Caa and sequence 2 is: cTA|CCT|Cag. Then we would calculate *K*_*s *_in PAML using the complete nine bases of the three codons. This approach modifies the data to a suitable form for analysis by PAML, at the expense of introducing sequence noise. To make use of all codons relevant to a motif, one calculates the *L*_*i*_, S_i_, and *V*_*i *_values by summing over all *n *codons that overlap any instance of the motif, where these codons are indexed by the variable *j*, that is:

#### Nucleotide-by-nucleotide method

To avoid the noise introduced by appending partial codons in the naïve codon completion method, we refined the method to compute the synonymous substitution rate on a nucleotide-by-nucleotide basis. The algorithm has a close analogy with the naïve codon completion method, with the parameters again given by the formulas:

However, here the index *j *is considered over all nucleotides overlapping the motifs, and *n *is equal to the number of nucleotides overlapping the motif. PAML cannot handle this type of sequence input due to the irregular translation frames of the nucleotides overlapping the motif. Therefore, we implemented the algorithm independently.

### Comparison to splicing motif experiments

ESE data for Figure 5 were obtained from Figure 4 of [4]. The values shown are equal to the splicing inclusion rates in the Fairbrother *et al*. [4] data rounded to the nearest 5%. Splicing inclusion and silencing rates for the Zhang and Chasin experiments were obtained from Figure 4 of [23].

### ACS motif

We identified a degenerate consensus sequence of TKTTTATRTTTWGT from the ACS motif logo of Figure 1B.iv reported in [25], where K = T/G, R = A/G, and W = T/A. We tested the z-scores of all hexamers consistent with this degenerate sequence.

### MicroRNA binding motifs

Rice and *Arabidopisis *microRNAs were obtained from miRBase [58]. Since not all of these have known seed sequences, every 8-mer aligned consistently across the species within these microRNAs was identified. The reverse complements of these 8-mers were then used for the set of potential plant microRNA binding sites (305 8-mers). For the animal analysis, motifs in Tables S1, S2, and S3 of [28] were used as a set of curated 7-mer microRNA seed sequences. The reverse complements of these 156 7-mers were analyzed. Note that the better available animal dataset is responsible for the higher μ of animal binding sites relative to plant, reported in the main text.

### CpG correction

For the CpG-modified z-scores, we assumed that the CpG effect was so strong that the expected frame-specific conservation rate at a CpG site would be independent of the amino acid sequence. That is, we first calculated the conservation rate of CpG dinucleotides occurring in each of the three codon frames (1.2), (2.3), and (3.1), respectively. We then incorporated these rates into the calculations of the expected number of conserved copies for each motif. For each instance of a motif containing a CpG, the expected conservation rate at those CpG positions was forced to be the frame-specific CpG conservation rate, as opposed to the rate that would be expected from the aligned amino acids.

## Abbreviations

COMIT: Coding Motif Identification Tool; ESE: exonic splicing enhancer; ESS: exonic splicing silencer.

## Authors' contributions

DK implemented the COMIT algorithm, analyzed data, helped design experiments, and contributed to the drafting of the manuscript. YD performed the microRNA analysis, analyzed data, helped design experiments, and contributed to the drafting of the manuscript. JW implemented the synonymous rate algorithms, analyzed data, and contributed to the drafting of the manuscript. AMK contributed to the dataset assembly, helped implement COMIT, and analyzed data. JHC conceived and coordinated the study and finalized the manuscript. All authors read and approved the final manuscript.

## Additional data files

The following additional data are available with the online version of this paper: a PDF containing Figures S1, S2, and S3 (Additional data file 1). a spreadsheet containing Tables S1, S2, and S3 (Additional data file 2).

## Supplementary Material

Additional data file 1 Figure S1: improved correlation in z-score and *K*_*s *_for larger clades. Figure S2: comparison of naïve codon completion and nucleotide-by-nucleotide *K*_*s *_methods. Figure S3: comparison of mouse-human COMIT scores to scores uncalibrated for amino acids. Click here for file

Additional data file 2 COMIT scores and nucleotide-by-nucleotide *K*_*s *_values for hexamers. Table S1: COMIT scores for all hexamers in multiple lineage comparisons. Table S2: *K*_*s *_values. Table S3: motifs with z > 15 in all three mammalian lineages. Click here for file

## References

[B1] Chamary JV Parmley JL Hurst LD Hearing silence: non-neutral evolution at synonymous sites in mammals. Nat Rev Genet 2006 7 98 108 10.1038/nrg1770 16418745

[B2] Jambhekar A DeRisi JL Cis-acting determinants of asymmetric, cytoplasmic RNA transport. RNA 2007 13 625 642 10.1261/rna.262607 17449729 PMC1852811

[B3] Sharp PM Li WH The codon Adaptation Index - a measure of directional synonymous codon usage bias, and its potential applications. Nucleic Acids Res 1987 15 1281 1295 10.1093/nar/15.3.1281 3547335 PMC340524

[B4] Fairbrother WG Yeh R-F Sharp PA Burge CB Predictive identification of exonic splicing enhancers in human genes. Science 2002 297 1007 1013 10.1126/science.1073774 12114529

[B5] Kudla G Lipinski L Caffin F Helwak A Zylicz M High guanine and cytosine content increases mRNA levels in mammalian cells. PLoS Biol 2006 4 e180 10.1371/journal.pbio.0040180 16700628 PMC1463026

[B6] Nackley AG Shabalina SA Tchivileva IE Satterfield K Korchynskyi O Makarov SS Maixner W Diatchenko L Human catechol-O-methyltransferase haplotypes modulate protein expression by altering mRNA secondary structure. Science 2006 314 1930 1933 10.1126/science.1131262 17185601

[B7] Kimchi-Sarfaty C Oh JM Kim I-W Sauna ZE Calcagno AM Ambudkar SV Gottesman MM A "silent" polymorphism in the MDR1 gene changes substrate specificity. Science 2007 315 525 528 10.1126/science.1135308 17185560

[B8] Itzkovitz S Alon U The genetic code is nearly optimal for allowing additional information within protein-coding sequences. Genome Res 2007 17 405 412 10.1101/gr.5987307 17293451 PMC1832087

[B9] Brodersen P Voinnet O Revisiting the principles of microRNA target recognition and mode of action. Nat Rev Mol Cell Biol 2009 10 141 10.1038/nrm2619 19145236

[B10] Anderson P Kedersha N RNA granules: post-transcriptional and epigenetic modulators of gene expression. Nat Rev Mol Cell Biol 2009 10 430 10.1038/nrm2694 19461665

[B11] Besse F Ephrussi A Translational control of localized mRNAs: restricting protein synthesis in space and time. Nat Rev Mol Cell Biol 2008 9 971 10.1038/nrm2548 19023284

[B12] Graur D Li W-H Fundamentals of Molecular Evolution 2000 2 Sunderland, MA: Sinauer

[B13] Chuang J Li H Similarity of synonymous substitution rates across mammalian genomes. J Mol Evol 2007 65 236 10.1007/s00239-007-9008-x 17674075

[B14] Cliften P Sudarsanam P Desikan A Fulton L Fulton B Majors J Waterston R Cohen BA Johnston M Finding functional features in *Saccharomyces *genomes by phylogenetic footprinting. Science 2003 301 71 76 10.1126/science.1084337 12775844

[B15] Xie X Lu J Kulbokas EJ Golub TR Mootha V Lindblad-Toh K Lander ES Kellis M Systematic discovery of regulatory motifs in human promoters and 3' UTRs by comparison of several mammals. Nature 2005 434 338 345 10.1038/nature03441 15735639 PMC2923337

[B16] MacIsaac KD Fraenkel E Practical strategies for discovering regulatory DNA sequence motifs. PLoS Comput Biol 2006 2 e36 10.1371/journal.pcbi.0020036 16683017 PMC1447654

[B17] Forman JJ Legesse-Miller A Coller HA A search for conserved sequences in coding regions reveals that the let-7 microRNA targets Dicer within its coding sequence. Proc Natl Acad Sci USA 2008 105 14879 14884 10.1073/pnas.0803230105 18812516 PMC2567461

[B18] Goren A Ram O Amit M Keren H Lev-Maor G Vig I Pupko T Ast G Comparative analysis identifies exonic splicing regulatory sequences - the complex definition of enhancers and silencers. Mol Cell 2006 22 769 10.1016/j.molcel.2006.05.008 16793546

[B19] Odom DT Dowell RD Jacobsen ES Gordon W Danford TW MacIsaac KD Rolfe PA Conboy CM Gifford DK Fraenkel E Tissue-specific transcriptional regulation has diverged significantly between human and mouse. Nat Genet 2007 39 730 10.1038/ng2047 17529977 PMC3797512

[B20] Prabhakar S Visel A Akiyama JA Shoukry M Lewis KD Holt A Plajzer-Frick I Morrison H FitzPatrick DR Afzal V Pennacchio LA Rubin EM Noonan JP Human-specific gain of function in a developmental enhancer. Science 2008 321 1346 1350 10.1126/science.1159974 18772437 PMC2658639

[B21] Li W-H Unbiased estimation of the rates of synonymous and nonsynonymous substitution. J Mol Evol 1993 36 96 10.1007/BF02407308 8433381

[B22] Yang Z PAML: a program package for phylogenetic analysis by maximum likelihood. Comput Appl Biosci 1997 13 555 556 9367129 10.1093/bioinformatics/13.5.555

[B23] Zhang XHF Chasin LA Computational definition of sequence motifs governing constitutive exon splicing. Genes Dev 2004 18 1241 1250 10.1101/gad.1195304 15145827 PMC420350

[B24] Wang Z Rolish ME Yeo G Tung V Mawson M Burge CB Systematic identification and analysis of exonic splicing silencers. Cell 2004 119 831 10.1016/j.cell.2004.11.010 15607979

[B25] Nieduszynski CA Knox Y Donaldson AD Genome-wide identification of replication origins in yeast by comparative genomics. Genes Dev 2006 20 1874 1879 10.1101/gad.385306 16847347 PMC1522085

[B26] Kellis M Patterson N Endrizzi M Birren B Lander ES Sequencing and comparison of yeast species to identify genes and regulatory elements. Nature 2003 423 241 254 10.1038/nature01644 12748633

[B27] Rigoutsos I New tricks for animal microRNAs: targeting of amino acid coding regions at conserved and nonconserved sites. Cancer Res 2009 69 3245 3248 10.1158/0008-5472.CAN-09-0352 19351814

[B28] Friedman RC Farh KK-H Burge CB Bartel DP Most mammalian mRNAs are conserved targets of microRNAs. Genome Res 2009 19 92 105 10.1101/gr.082701.108 18955434 PMC2612969

[B29] Ke S Zhang XHF Chasin LA Positive selection acting on splicing motifs reflects compensatory evolution. Genome Res 2008 18 533 543 10.1101/gr.070268.107 18204002 PMC2279241

[B30] Parmley JL Chamary JV Hurst LD Evidence for purifying selection against synonymous mutations in mammalian exonic splicing enhancers. Mol Biol Evol 2006 23 301 309 10.1093/molbev/msj035 16221894

[B31] Zhang XHF Leslie CS Chasin LA Computational searches for splicing signals. Methods 2005 37 292 10.1016/j.ymeth.2005.07.011 16314258

[B32] Yeo G Hoon S Venkatesh B Burge CB Variation in sequence and organization of splicing regulatory elements in vertebrate genes. Proc Natl Acad Sci USA 2004 101 15700 15705 10.1073/pnas.0404901101 15505203 PMC524216

[B33] Stadler MB Shomron N Yeo GW Schneider A Xiao X Burge CB Inference of splicing regulatory activities by sequence neighborhood analysis. PLoS Genet 2006 2 e191 10.1371/journal.pgen.0020191 17121466 PMC1657047

[B34] Itoh H Washio T Tomita M Computational comparative analyses of alternative splicing regulation using full-length cDNA of various eukaryotes. RNA 2004 10 1005 1018 10.1261/rna.5221604 15208437 PMC1370592

[B35] Kim Guisbert KS Li H Guthrie C Alternative 3' pre-mRNA processing in *Saccharomyces cerevisiae *is modulated by Nab4/Hrp1 *in vivo*. PLoS Biol 2007 5 e6 10.1371/journal.pbio.0050006 17194212 PMC1717019

[B36] Olivier C Poirier G Gendron P Boisgontier A Major F Chartrand P Identification of a conserved RNA motif essential for She2p recognition and mRNA localization to the yeast bud. Mol Cell Biol 2005 25 4752 4766 10.1128/MCB.25.11.4752-4766.2005 15899876 PMC1140632

[B37] Hogan DJ Riordan DP Gerber AP Herschlag D Brown PO Diverse RNA-binding proteins interact with functionally related sets of RNAs, suggesting an extensive regulatory system. PLoS Biol 2008 6 e255 10.1371/journal.pbio.0060255 18959479 PMC2573929

[B38] Down T Leong B Hubbard T A machine learning strategy to identify candidate binding sites in human protein-coding sequence. BMC Bioinformatics 2006 7 419 10.1186/1471-2105-7-419 17002805 PMC1592515

[B39] Robins H Krasnitz M Levine AJ The computational detection of functional nucleotide sequence motifs in the coding regions of organisms. Exp Biol Med (Maywood) 2008 233 665 673 10.3181/0704-MR-97 18408149

[B40] Chen H Blanchette M Detecting non-coding selective pressure in coding regions. BMC Evol Biol 2007 7 S9 10.1186/1471-2148-7-S1-S9 17288582 PMC1796618

[B41] Schattner P Diekhans M Regions of extreme synonymous codon selection in mammalian genes. Nucleic Acids Res 2006 34 1700 1710 10.1093/nar/gkl095 16556911 PMC1410912

[B42] Siepel A Bejerano G Pedersen JS Hinrichs AS Hou M Rosenbloom K Clawson H Spieth J Hillier LW Richards S Weinstock GM Wilson RK Gibbs RA Kent WJ Miller W Haussler D Evolutionarily conserved elements in vertebrate, insect, worm, and yeast genomes. Genome Res 2005 15 1034 1050 10.1101/gr.3715005 16024819 PMC1182216

[B43] Chuang JH Li H Functional bias and spatial organization of genes in mutational hot and cold regions in the human genome. PLoS Biol 2004 2 E29 10.1371/journal.pbio.0020029 14966531 PMC340940

[B44] Drummond DA Wilke CO Mistranslation-induced protein misfolding as a dominant constraint on coding-sequence evolution. Cell 2008 134 341 10.1016/j.cell.2008.05.042 18662548 PMC2696314

[B45] Siepel A Haussler D Phylogenetic estimation of context-dependent substitution rates by maximum likelihood. Mol Biol Evol 2004 21 468 488 10.1093/molbev/msh039 14660683

[B46] Majoros WH Ohler U Complexity reduction in context-dependent DNA substitution models. Bioinformatics 2009 25 175 182 10.1093/bioinformatics/btn598 19017657 PMC2732293

[B47] Baele G Peer Y Van de Vansteelandt S A model-based approach to study nearest-neighbor influences reveals complex substitution patterns in non-coding sequences. Syst Biol 2008 57 675 692 10.1080/10635150802422324 18853356

[B48] Gunewardena S Zhang Z A hybrid model for robust detection of transcription factor binding sites. Bioinformatics 2008 24 484 491 10.1093/bioinformatics/btm629 18184687

[B49] Guo HH Choe J Loeb LA Protein tolerance to random amino acid change. Proc Natl Acad Sci USA 2004 101 9205 9210 10.1073/pnas.0403255101 15197260 PMC438954

[B50] Parmley JL Urrutia AO Potrzebowski L Kaessmann H Hurst LD Splicing and the evolution of proteins in mammals. PLoS Biol 2007 5 e14 10.1371/journal.pbio.0050014 17298171 PMC1790955

[B51] Parmley JL Hurst LD Exonic splicing regulatory elements skew synonymous codon usage near intron-exon boundaries in mammals. Mol Biol Evol 2007 24 1600 1603 10.1093/molbev/msm104 17525472

[B52] Warnecke T Batada NN Hurst LD The impact of the nucleosome code on protein-coding sequence evolution in yeast. PLoS Genet 2008 4 e1000250 10.1371/journal.pgen.1000250 18989456 PMC2570795

[B53] Washietl S Machné R Goldman N Evolutionary footprints of nucleosome positions in yeast. Trends Genet 2008 24 583 10.1016/j.tig.2008.09.003 18951646

[B54] Fox A Tuch B Chuang J Measuring the prevalence of regional mutation rates: an analysis of silent substitutions in mammals, fungi, and insects. BMC Evol Biol 2008 8 186 10.1186/1471-2148-8-186 18588686 PMC2447844

[B55] Chin CS Chuang JH Li H Genome-wide regulatory complexity in yeast promoters: separation of functionally conserved and neutral sequence. Genome Res 2005 15 205 213 10.1101/gr.3243305 15653830 PMC546519

[B56] TIGR ftp ftp://ftp.tigr.org/

[B57] Imamura H Persampieri J Chuang J Sequences conserved by selection across mouse and human malaria species. BMC Genomics 2007 8 372 10.1186/1471-2164-8-372 17937810 PMC2174483

[B58] Griffiths-Jones S Saini HK van Dongen S Enright AJ miRBase: tools for microRNA genomics. Nucleic Acids Res 2008 36 D154 158 10.1093/nar/gkm952 17991681 PMC2238936

