# Supplementary material for: COMIT: identification of noncoding motifs under selection in coding sequences
Source: Genome Biol. 2009 Nov 20;10(11):R133. doi: 10.1186/gb-2009-10-11-r133 (PMC3091326; doi:10.1186/gb-2009-10-11-r133)
Supplement: Additional data file 1 — Figure S1: improved correlation in z-score and Ks for larger clades. Figure S2: comparison of naïve codon completion and nucleotide-by-nucleotide Ks methods. Figure S3: comparison of mouse-human COMIT scores to scores uncalibrated for amino acids. [file gb-2009-10-11-r133-S1.PDF]

Figure S1. Improved Correlation in z-score and K_s for Larger Clades

A) For each of the 4^6 possible 6-mers, we calculated the total K_s tree length in the (human, (dog, (rat, mouse))) phylogeny, as shown in the figure below (Note K_s is labeled as dS in the figure).

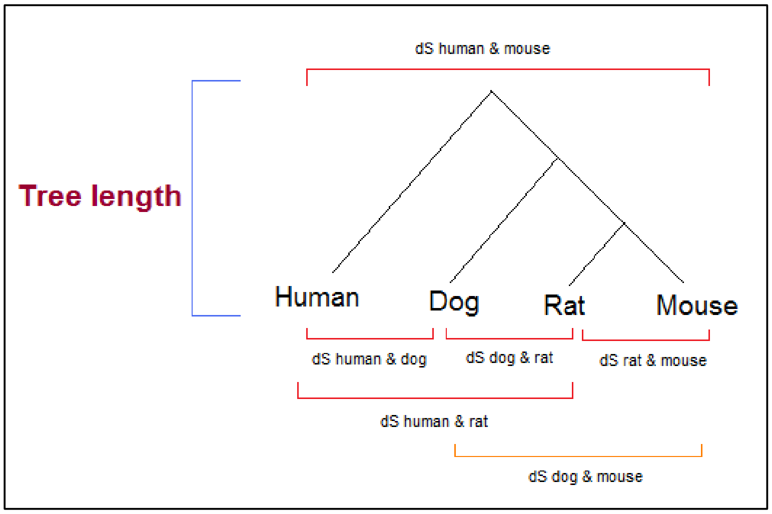

Instances of the motif were defined based on their occurrence in the human coding region. K_s values were calculated pairwise and these were then aggregated to determine the total K_s of the phylogeny, according to the formula:

B) Tree length=[K_s (dog-mouse)+ K_s (human-rat)+ K_s (human-dog)+ K_s (rat-mouse)]/2
The figure below shows a plot of this tree length versus the human-mouse conservation z-score. There is a strong negative correlation between these two measures, even stronger than that between K_s (human-mouse) and the human-mouse conservation z-score. This is because the K_s method gains resolving power with increasing number of species, allowing a better comparison to the z-score approach.

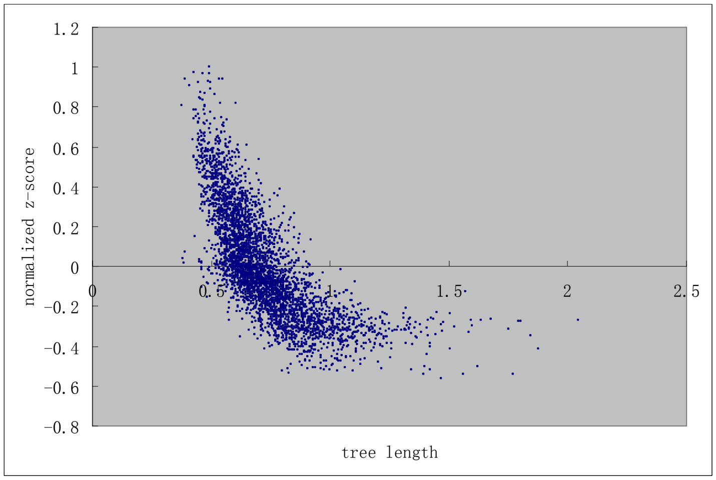

Figure S2. Comparison of Naïve Codon Completion and Nucleotide-by-Nucleotide K_s Methods

Tree length=[K_s (dog-mouse)+ K_s (human-rat)+ K_s (human-dog)+ K_s (rat-mouse)]/2

Using the above formula we calculated the total tree length for each 6-mer for both the nucleotide-by-nucleotide and naïve codon completion K_s methods. The distribution of K_s values for each method is shown below. The nucleotide-by-nucleotide K_s distribution (red curve) has generally smaller K_s values than that for the naïve codon completion K_s distribution (blue curve). This is consistent with the naive method being more subject to noise. The naïve K_s distribution is also broader, for the same reason. The narrowness of the nucleotide-by-nucleotide K_s distribution makes that method better for detecting outlier K_s values than the naïve K_s method. However, the COMIT z-score method is superior to either of the K_s methods for detecting unusually conserved motifs.

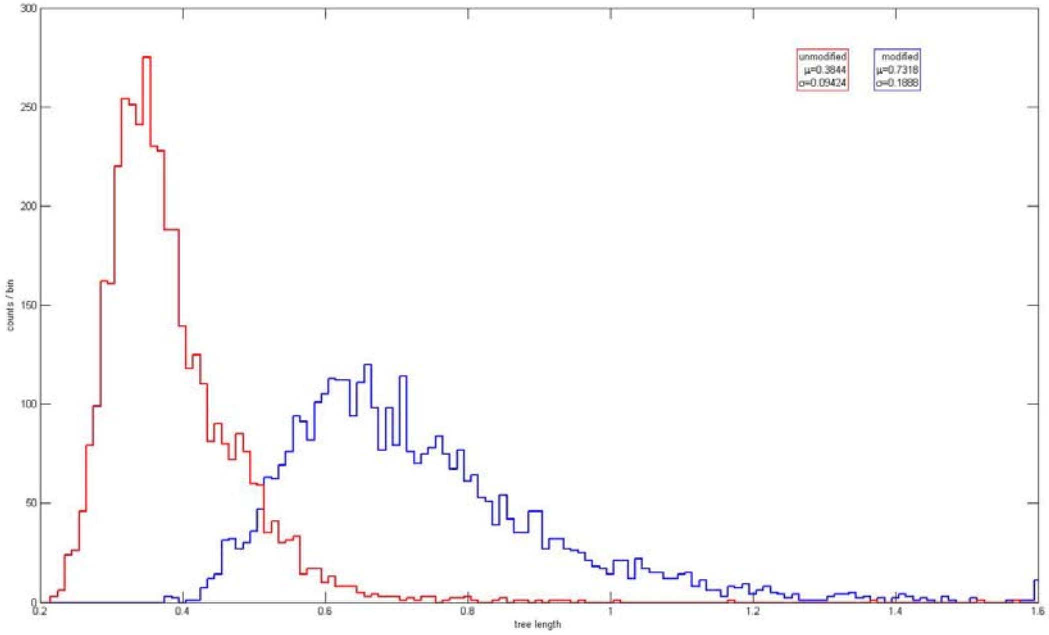

Figure S3. Correlation of Mouse-Human COMIT Scores to Scores Uncalibrated for Amino Acid.

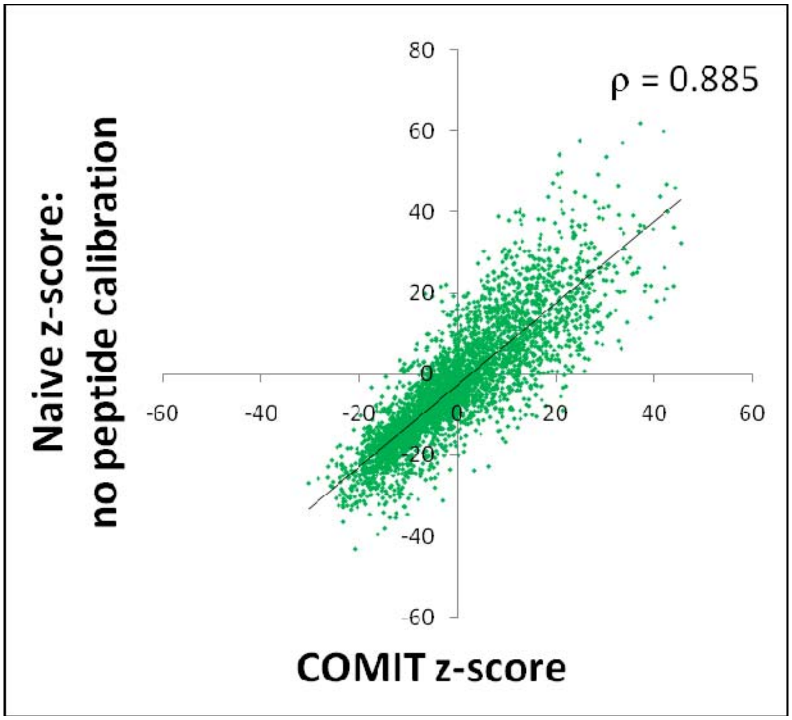

Scores are calculated from comparisons of mouse and human coding regions. The correlation has Spearman ρ = 0.885 ($p<0.00001$, Permutation test). Using a Pearson correlation, R^2 = 0.7349 ($p<1e-300$, t-test). When only those motifs with COMIT score > 0 are considered, the correlation has R^2 = 0.3989 ($p<1e-187$, t-test).
